# Investigation of the Chemical Structure of Ultra-Thin Polyimide Substrate for the Xenon Flash Lamp Lift-off Technology

**DOI:** 10.3390/polym13040546

**Published:** 2021-02-12

**Authors:** Seong Hyun Jang, Young Joon Han, Sang Yoon Lee, Geonho Lee, Jae Woong Jung, Kwan Hyun Cho, Jun Choi

**Affiliations:** 1 Human Convergence Technology R&D Department, Korea Institute of Industrial Technology (KITECH), Ansan 15588, Korea; seonghyun@kitech.re.kr (S.H.J.); sangyoon@kitech.re.kr (S.Y.L.); geonho118@kitech.re.kr (G.L.); 2 Department of Advanced Material Engineering for Information & Electrices, Kyunghee University, Yongin 17104, Korea; 3 Manufacturing Process Platform R&D Department, Korea Institute of Industrial Technology (KITECH), Ansan 15588, Korea; youngjhan@kitech.re.kr; 4 Department of Chemical Engineering, Kyunghee University, Yongin 17104, Korea

**Keywords:** polyimide, lift-off, xenon flash lamp, chemical structure, colorless, flexible electronics

## Abstract

Lift-off is one of the last steps in the production of next-generation flexible electronics. It is important that this step is completed quickly to prevent damage to ultrathin manufactured electronics. This study investigated the chemical structure of polyimide most suitable for the Xe Flash lamp–Lift-Off process, a next-generation lift-off technology that will replace the current dominant laser lift-off process. Based on the characteristics of the peeled-off polyimide films, the Xe Flash lamp based lift-off mechanism was identified as photothermal decomposition. This occurs by thermal conduction via light-to-heat conversion. The synthesized polyimide films treated with the Xe Flash lamp–Lift-Off process exhibited various thermal, optical, dielectric, and surface characteristics depending on their chemical structures. The polyimide molecules with high concentrations of –CF_3_ functional groups and kinked chemical structures demonstrated the most promising peeling properties, optical transparencies, and dielectric constants. In particular, an ultra-thin polyimide substrate (6 μm) was successfully fabricated and showed potential for use in next-generation flexible electronics.

## 1. Introduction

Flexible electronics, such as foldable, rollable, and wearable devices, are receiving attention for their ability to maintain the performance of existing flat electronics while transforming to suit the environment of the user [1,2]. One of the most important technologies used in flexible electronics is substrate formation including lift-off process. Research is currently being conducted on the best methods to compose polymer substrates, such as polyethylene terephthalate (PET), polyethylene naphthalate (PEN), polycarbonate (PC), polyestersulfone (PES), and polyimide (PI) [3,4,5,6,7]. It has been determined that PI, which has extremely high heat and chemical resistance, practical mechanical properties, and advantageous insulating properties, is suitable as a substrate component of high-technology electronic devices. Because of these characteristics, transparent PI films, which reduce the charge transfer complexes (CTCs) between intra- and inter-chain systems, have the potential to act as electrical multilayer components in flexible displays and solar cells [8,9].

Flexible electronics can be manufactured using a PI substrate according to the following method. First, a PI film is fabricated on a rigid carrier substrate. Next, electrical multilayer components (such as TFTs and photoactive layers) are fabricated and subsequently sealed via a packaging process. Finally, the whole device, including the flexible PI substrate, is detached from the carrier substrate. The final step, the laser lift-off (LLO) process, is one of the key techniques of flexible electronics manufacturing and determines the production speed and yield of the process [10,11,12].

The current market-dominating LLO process uses lasers, which brings about many disadvantages. First, the equipment investment, maintenance, and repairs costs are considerable because conventional excimer lasers require expensive high-precision optics and energy sources, such as XeCl and KrF. Second, the LLO process operates according to the line scan method, which accumulates many single irradiation pathways and slows down production. Using this method, the peeled-off film can also accumulate deformations along the scan lines, causing final device deterioration. Lastly, this method is limited in its ability to reduce the thickness of the substrate to increase device flexibility. It is costly and time-consuming to realize an ultrathin substrate because it requires a very low fluence of the excimer laser and multiple scans to achieve the desired result. Overall, the LLO method needs many improvements to minimize cost, time, and substrate thickness.

Studies of the lift-off method using PI films were conducted in the mid-1980s, starting with investigations of the laser ablation of various polymer materials in 1978 [13,14,15,16]. In the last three years, studies of ultrathin films for advanced flexible electronics have increased. Bian et al. [10] reported that lowering the excimer laser fluence and increasing the number of scans reduces the thickness of polyimide film during the LLO process. Lee et al. [17] reported a new lift-off process using a Xe Flash lamp power source and a specially developed light-to-heat conversion (LTHC) layer. At this time, no studies have reported the investigations of differently structured polymer substrates in the new lift-off process.

In this study, the lift-off behavior of PI films with various chemical structures was investigated for use in the new “Xe Flash lamp–Lift-Off” (XF-LO) process. The PI substrates used in the experiment were designated as non-fluorine polyimide (BPI series) or fluorine polyimide (FPI series), and synthesized in a manner suitable to their chemical structures. The film thickness was varied by controlling the viscosity of the poly (amic acid) (PAA), and the surface peeling behavior at various film thicknesses was observed. The structural arrangements, heat resistances, optical properties, and dielectric properties of the peeled-off films were then evaluated. In particular, the surface characteristics of the released films were closely examined.

## 2. Materials and Methods

### 2.1. Materials and Instruments

The compounds pyromellitic dianhydride (PMDA); 4,4′-biphthalic anhydride (s-BPDA); 4,4′-(hexafluoroisopropylidene)diphthalic anhydride (6FDA); 1,4-phenylenediamine (PDA); 4,4′-diaminnodiphenyl ether (ODA); 4,4′-oxibis [3-(trifluoromethyl)aniline] (6FODA); and 2,2′-bis(trifluoromethyl)benzidine (TFDB) were purchased from TCI, Tokyo, Japan. Dimethylacetamide (DMAc) was obtained from Samchun Chemicals, Seoul, Korea. Viscosities were measured with a Brookfield Programmable Digital Viscometer DV- II + Pro LV from Brookfield engineering laboratories, Inc., MA, USA. Thicknesses of the peeled-off films were measured with a Mitutoyo ABS Digimatic Indicator ID-C125XB from Mitutoyo, Tokyo, Japan. Fourier-Transform Infrared Spectroscopy (FT-IR) was measured using a Thermo Fisher Scientific Nicolet iS50 FTIR Spectrometer from MA, USA. Field emission scanning electron microscopy (FE-SEM) images were collected on a HITACHI SU8000 from Tokyo, Japan. Energy-dispersive X-ray spectroscopy (EDS) analysis data were collected on a Thermo Fisher Pathfinder from MA, USA. Thermogravimetric analysis (TGA) was conducted under nitrogen at a heating rate of 20 °C/min using a TA Instruments Thermogravimetric Analyzer Q500 from DE, USA. Absorption spectra were measured using a Scinco Mega-800 UV/Vis spectrometer from Seoul, Korea. Apparent contact angles were measured using an SEO’s Contact Angle Meter Phoenix-MT(T) from Suwon, Korea. Dielectric constants were collected from 100 kHz to 13 MHz using an Agilent LCR Meter from CA, USA.

### 2.2. Synthesis of Poly(Amic Acid) Solutions

PAA solutions were synthesized separately for BPI series (non CF_3_ contents) and FPI series (–CF_3_ contents). The viscosities of all PAA solutions were optimized to 10,000–11,000 cP.

#### 2.2.1. BPI (BPDA-PDA) (BPI-a)

The compounds PDA (4.1 g, 0.038 mol) and DMAc (85 g, 90.4 mL) were placed in the reaction vessel and stirred with a mechanical stirrer. Subsequently, powdered BPDA (10.9 g, 0.037 mol) was slowly added to the mixture. The reaction vessel was then sealed, and nitrogen gas was circulated inside the vessel. The reaction vessel was placed in an ice bath for 2 h. After this time, the vessel was removed from the ice bath and stirred at room temperature for 4 h. Finally, the PAA solution was obtained from the vessel. The viscosity was 10,000 cP. The Fourier-Transform Infrared Spectroscopy (FT-IR) of the corresponding imidized film was as follows, FT-IR (neat): *v* (cm^−1^) 1720 (C=O), 1489 (C=C), 1392 (C–N–C).

#### 2.2.2. BPI (PMDA-ODA) (BPI-b)

The compounds ODA (7.2 g, 0.036 mol) and DMAc (85 g, 90.4 mL) were placed in the reaction vessel and stirred with a mechanical stirrer. Subsequently, powdered PMDA (7.8 g, 0.036 mol) was slowly added to the mixture. The reaction vessel was then sealed, and nitrogen gas was circulated inside the vessel. The reaction vessel was placed in an ice bath for 2 h. Thereafter, the vessel was removed from the ice bath and stirred at room temperature for 4 h. Finally, the PAA solution was obtained from the vessel. The viscosity was 10,500 cP. The FT-IR of the corresponding imidized film was as follows, FT-IR (neat): *v* (cm^−1^) 1715 (C=O), 1492 (C=C), 1390 (C–N–C), 1160 (C–O–C).

#### 2.2.3. BPI (BPDA-ODA) (BPI-c)

The compounds ODA (6.9 g, 0.034 mol) and DMAc (83 g, 88.3 mL) were placed in the reaction vessel and stirred with a mechanical stirrer. Subsequently, powdered BPDA (10.1 g, 0.034 mol) was slowly added to the mixture. The reaction vessel was then sealed, and nitrogen gas was circulated inside the vessel. The reaction vessel was placed in an ice bath for 2 h. Thereafter, the vessel was removed from the ice bath and stirred at room temperature for 4 h. Finally, the PAA solution was obtained from the vessel. The viscosity was 10,500 cP. The FT-IR of the corresponding imidized film was as follows, FT-IR (neat): *v* (cm^−1^) 1721 (C=O), 1489 (C=C), 1391 (C–N–C), 1155 (C–O–C).

#### 2.2.4. FPI (PMDA-TFDB) (FPI-a)

The compounds TFDB (9 g, 0.028 mol) and DMAc (85 g, 90.4 mL) were placed in the reaction vessel and stirred with a mechanical stirrer. Subsequently, powdered PMDA (6.0 g, 0.028 mol) was slowly added to the mixture. The reaction vessel was then sealed, and nitrogen gas was circulated inside of the vessel. The reaction vessel was placed in an ice bath for 2 h. Thereafter, the vessel was removed from the ice bath and stirred at room temperature for 4 h. Finally, the PAA solution was obtained from the vessel. The viscosity was 11,000 cP. The FT-IR of the corresponding imidized film was as follows, FT-IR (neat): *v* (cm^−1^) 1725 (C=O), 1494 (C=C), 1386 (C–N–C), 1310 (C–F).

#### 2.2.5. FPI (PMDA-6FODA) (FPI-b)

The compounds 6FODA (9.1 g, 0.027 mol) and DMAc (85 g, 90.4 mL) were placed in the reaction vessel and stirred with a mechanical stirrer. Subsequently, powdered PMDA (5.9 g, 0.027 mol) was slowly added to the mixture. The reaction vessel was then sealed, and nitrogen gas was circulated inside of the vessel. The reaction vessel was placed in an ice bath for 2 h. Thereafter, the vessel was removed from the ice bath and stirred at room temperature for 4 h. Finally, the PAA solution was obtained from the vessel. The viscosity was 10,300 cP. The FT-IR of the corresponding imidized film was as follows, FT-IR (neat): *v* (cm^−1^) 1723 (C=O), 1495 (C=C), 1388 (C–N–C), 1311 (C–F), 1160 (C–O–C).

#### 2.2.6. FPI (6FDA-TFDB) (FPI-c)

The compounds TFDB (7.3 g, 0.023 mol) and DMAc (83 g, 88.3 mL) were placed in the reaction vessel and stirred with a mechanical stirrer. Subsequently, powdered 6FDA (9.7 g, 0.022 mol) was slowly added to the mixture. The reaction vessel was then sealed, and nitrogen gas was circulated inside of the vessel. The reaction vessel was placed in an ice bath for 2 h. Thereafter, the vessel was removed from the ice bath and stirred at room temperature for 4 h. Finally, the PAA solution was obtained from the vessel. The viscosity was 10,500 cP. The FT-IR of the corresponding imidized film was as follows, FT-IR (neat): *v* (cm^−1^) 1730 (C=O), 1493 (C=C), 1390 (C–N–C), 1310 (C–F).

### 2.3. Fabrication of Polyimide Films

Six kinds of synthesized PAA solutions were cast on Mo-LTHC carrier glass (25 × 25 mm^2^) by the spin-coating method at 3000 rpm and 120 s. The cast PAA samples were prebaked in an oven with nitrogen gas at 100 °C for 30 min. They were then cured at 200 °C for 8 min and 350 °C for 20 min. The imidized PI film cast on the Mo-LTHC carrier glass was left to cool down to room temperature.

### 2.4. XF-LO Process Applied to Polyimide Films

The XF-LO process used a Xe Flash lamp (XF 12100LCW, Unilam Co., Ltd., Ulsan, Korea) as a light source. The cured PI films on the LTHC carrier glass were placed on the stage and then triggered by XFL pulses. The applied XFL Energy was fixed at a pulse height of 500 V, and the exposure time was varied from 1–7 ms [17]. The thicknesses of released films were 12 μm.

## 3. Results and Discussion

### 3.1. XF-LO Process Behavior of the Synthesized Polyimide Films

The synthesized PAA samples were spin-coated onto a specially fabricated carrier glass with a Mo-LTHC layer, and the PI films were produced through a thermal curing process. The PI film ablation was performed using the XF-LO process. Light was irradiated onto the LTHC layer at 500 V, and the pulse width was adjusted to 1, 3, 5, and 7 ms. The resulting Xenon Flash Lamp (XFL) fluences were 0.85, 2.6, 4.2, and 5.8 J/cm^2^, respectively. The ablation mechanism of conventional LLO method is still under consideration. One possible mechanism is photochemical ablation, in which the chemical bonds of the material are broken by electron excitation. Another possible mechanism is photothermal ablation, which is governed by the bulk photothermal bond breakage and gasification of polymer materials [12]. It is known that the LTHC layer converts light energy to heat energy; thus, the driving force of the XF-LO process is considered to be the volumetric ablation and gasification of the polymer material by the photothermal mechanism.

**Figure 1:**
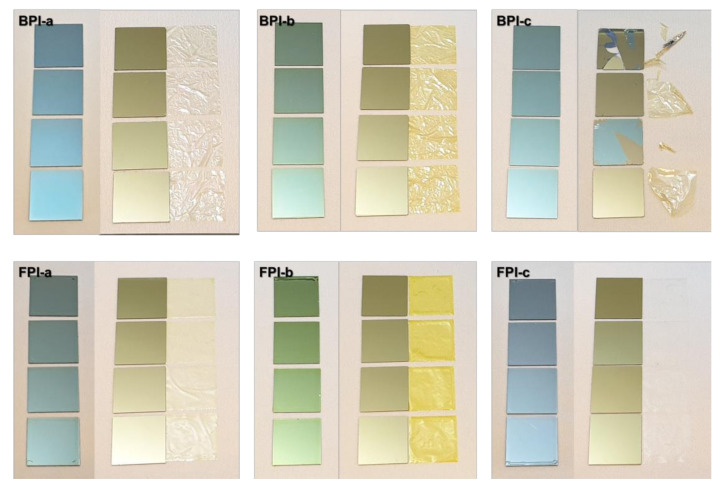
Ablation behavior of the prepared PI films before and after the XF-LO process. (The exposed XFL fluence, from top to bottom, were 0.85, 2.6, 4.2 and 5.8. J/cm^2^, respectively.).

**Scheme 1:**
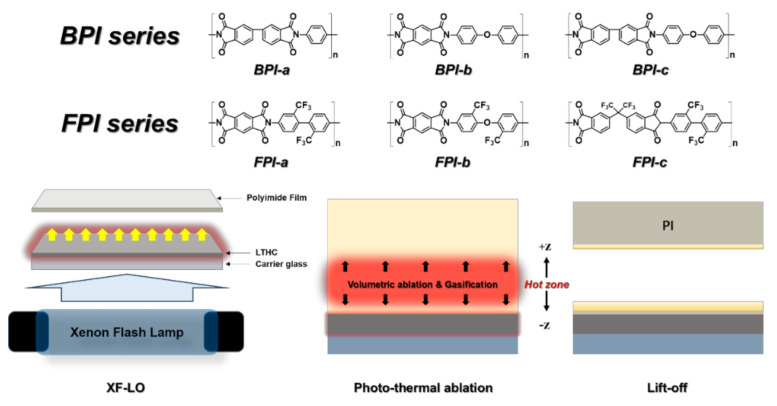
Chemical structures of prepared polyimide series and the XF-LO process mechanism.

Figure 1 shows the ablation behavior of the prepared PI films before and after the XF-LO process. The exposed XFL fluences were 0.85–5.8 J/cm^2^. After the XF-LO process, the LTHC looked yellowish due to carbonized PI that shed from the peeled film. It is believed that some of the PI molecules were cut off inside the film matrix by the XF-LO process. In internal ablation, heat is conducted from the LTHC to the PI matrix. The inner region of the PI close to the interface forms a hot zone, which is the temperature maxima region. Thermal decomposition and gasification progress toward the +z and –z axes in this hot zone, and the resulting gas pressure assists the lift-off process (Scheme 1).

**Figure 2:**
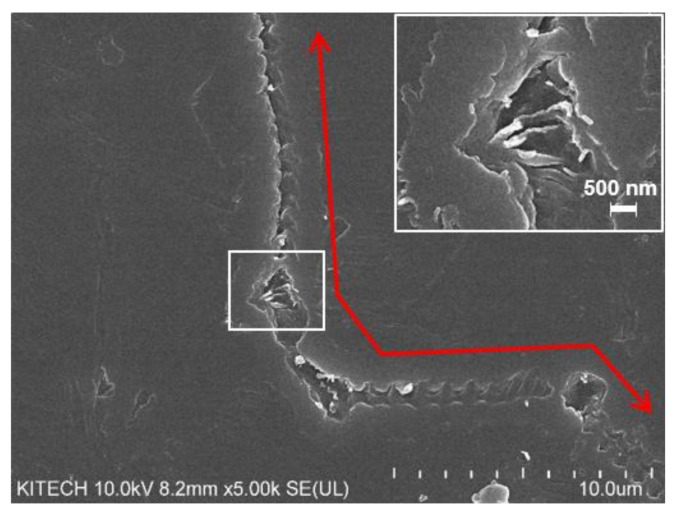
FE-SEM image of the BPI-a film after it was released from the surface.

The BPI-a and BPI-b films were peeled-off in the fluence range 0.85–5.8 J/cm^2^, but significant wrinkles occurred on the entire film surface. Figure 2 shows an FE-SEM image illustrating the BPI-a film with wrinkles. Various defects caused these wrinkles along the red auxiliary line. An enlarged image of the area with the largest defect shows aggregation aligned toward the horizontal axis. According to the references, structures such as BPI-a have been reported to have anisotropic linear thermal expansion. Because of this expansion, the serious wrinkles were caused on the plane. [18] The BPI-c film did not maintain its shape at any fluence and was shrunk or torn entirely. The reason for this is believed to be that BPI-c has the highest degree of freedom in its chemical structure among the BPI series. Its chemical composition contains a dianhydride moiety, which has a σ bond, and a diamine moiety, which has ether linkages. These structural elements can increase molecular rotation and bending, especially when exposed to heat, and can result in harsh deformations. The BPI-c sample was excluded from the successive measurements because it did not maintain the film shape.

Meanwhile, all FPI series, in which the –CF_3_ functional group was introduced into the diamine or dianhydride monomer, stayed without significant wrinkles when peeled-off according to the XF-LO process. This is likely due to the chemical structure of the FPI series. As the C–F bond is one of the strongest chemical bonds, fluorocarbon species such as –CF_3_ functional groups generally have unique properties including high surface activity and reliable thermal and chemical stability. [19] These properties likely allow the FPI series to be peeled-off without severe defects. In particular, FPI-c films substituted with –CF_3_ functional groups in both the diamine and dianhydride showed the most stable lift-off behavior over the entire applied XFL fluence range. As the presence of –CF_3_ functional groups in the molecule increased, the lift-off behavior and optical transparency also improved. Figure S1 shows FT-IR spectra and EDX data of the prepared FPI film series.

### 3.2. Investigation of Thermal Stability of Peeled-off Polyimide Films

**Figure 3:**
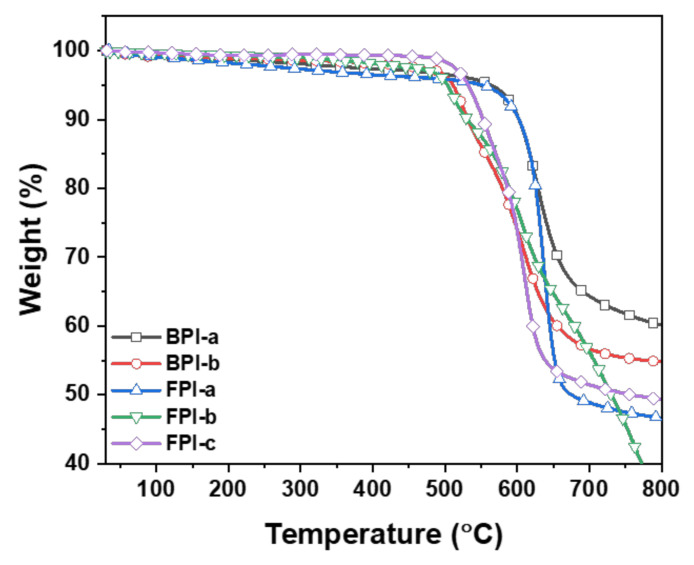
TGA curves of the prepared PI films.

Aromatic PIs have high heat resistance due to their strong bonding properties with intra- and inter-molecular CTCs [20]. The CTCs of the aromatic PIs become increasingly stable when the π electrons are transferred from the diamine (the electron donor) to the dianhydride (the electron acceptor). The intra- and inter-molecular bonds are strengthened due to this charge state of the chain, resulting in high heat resistance. Interestingly, the π electrons absorbed light in the visible region, which resulted in the yellowish color of the films [21]. To improve the optical transparency, the electron density was reduced with electro-withdrawing group such as fluorine or sulfone. However, these additions are known to reduce the thermal stability of PI [22]. Figure 3 shows the TGA curves of the prepared PI films peeled-off at the XFL fluence of 0.85 J/cm^2^. The prepared PI films showed high thermal stability up to 500 °C. All of the weight loss temperatures (T5 wt%) were over 500 °C, but the FPI series with –CF_3_ functional group showed the lower T5 wt% values. The BPI-b and FPI-b films, which had the same dianhydride as PMDA and diamine structures with ether linkages, had T5 wt% values of 510.9 °C and 503.1 °C, respectively. The BPI-a and FPI-a films, which were fully aromatic PIs without ether linkages in the main chain structures, had the higher T5 wt% values of 563.6 °C and 550.0 °C, respectively. The FPI-c film, which had the cleanest lift-off behavior without any damage or deformation, showed the best thermal stability up to 530 °C, but decomposed rapidly past 550 °C.

### 3.3. Investigation of Optical Properties of Peeled-off Polyimide Films

**Figure 4:**
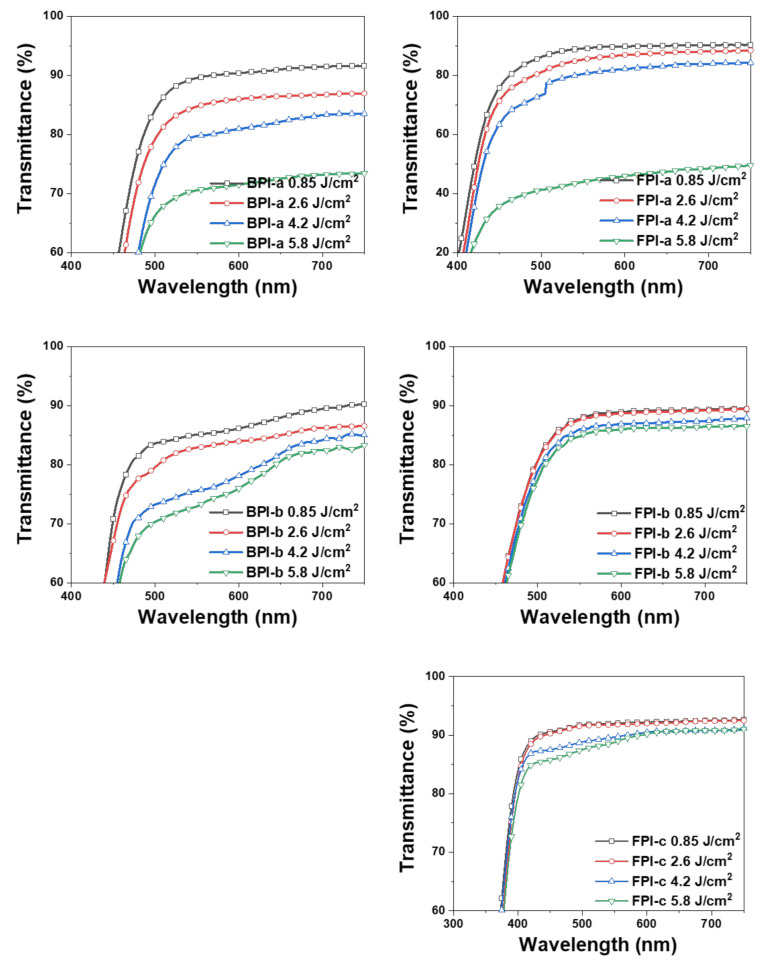
Optical transmittance curves of the peeled-off PI films with various applied XFL fluences.

Figure 4 and Table 1 show the light transmittance in the visible region of the peeled-off PI films for each applied XFL fluence. First, it was confirmed that the transmittance of the BPI-a and BPI-b films decreases as the XFL fluence increases, resulting in a weak yellow color. The mean transmittances of these films were measured from 500 to 750 nm. As the XFL fluence increased, the transmittance of BPI-a and BPI-b decreased from 90.2% to 71.6% and from 87.1% to 77.8%, respectively. Because the chemical structures of the BPI series do not contain fluorine-based functional groups, as shown in Figure 1, the XF-LO process caused damage, including fine wrinkles. It is believed that the damage to the material intensified as the XFL fluence increased, resulting in decreased transmittance. The transmittance of the FPI-a film also decreased as the applied XFL fluence increased. As the fluence increased from 0.85 to 4.2 J/cm^2^, its mean transmittance decreased slightly from 89.5% to 82.1%. When exposed to a fluence of 5.8 J/cm^2^, the mean transmittance decreased drastically to 46.2%. While there were no serious wrinkles in the FPI-a sample, a considerable haze formed on the film surface during the XF-LO process. 

According to a previous study, haze occurs when light is scattered by the film as it passes through the film [23]. These kinds of hazes in PI films are generated by the agglomeration and lamellar structure formation of polycrystalline three-dimensional structures during the chain-chain interactions promoted by heat energy [24]. The chain structures with kinked linkages or bulky substituents, such as –CF_3_ functional groups, which exhibit anti-aggregation tendencies, are known to be effective at inhibiting haze formation by heat. The FPI-a molecule, with a rigid rod structure, has the lowest degree of freedom among the FPI series and is expected to have the highest probability of inter-chain aggregation due to high level XFL heat energy. However, serious deterioration did not appear in the low fluence range.

As the XFL fluence increased, the FPI-b and FPI-c films, which have kinked structures and –CF_3_ functional groups, peeled-off without notable decreases in transmittance. Therefore, the damage to the surface caused by heat conduction through the LTHC was small. While the FPI-b sample did not transmit under the 450 nm range, the FPI-c sample with the highest –CF_3_ content showed the highest average optical transmittance, as shown in Table 1.

### 3.4. Investigation of Film Characteristics According to Thickness

**Figure 5:**
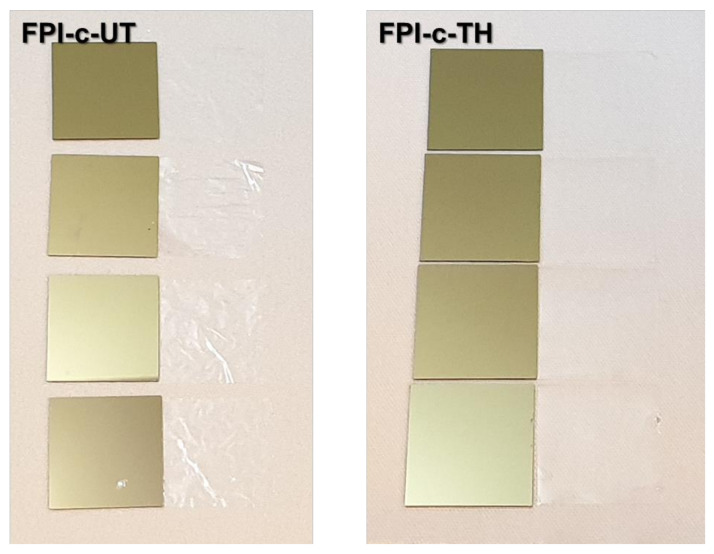
Ablation behavior of the FPI-c-UT and FPI-c-TH films after the XF-LO process. (The exposed XFL fluence, from top to bottom, were 0.85, 2.6, 4.2 and 5.8 J/cm^2^, respectively.).

**Figure 6:**
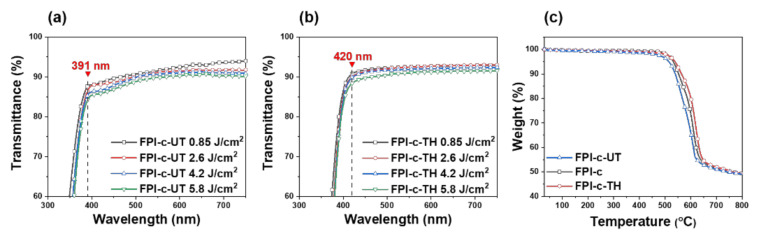
Optical transmittance curves of the peeled-off (**a**) FPI-c-UT and (**b**) FPI-c-TH films with various applied XFL fluences and (**c**) TGA curves of the prepared FPI-c film series.

After the XF-LO process, the characteristics of the peeled-off films were evaluated with respect to their thicknesses. The FPI-c sample, which showed the most stable lift-off characteristics, was selected for this experiment. In this experiment, the viscosity of the PAA solution was increased from 4500 cP and 25,000 cP, corresponding to solid contents of 13 wt% and 20 wt%, respectively. The thicknesses of the peeled-off films after imidization and the XF-LO process were 6 μm (FPI-c-UT) and 30 μm (FPI-c-TH), respectively. Figure 5 shows FPI-c-UT and FPI-c-TH after fabrication. The FPI-c-UT sample did not show noticeable yellowing or physical damage despite its thinness. The thicker FPI-c-TH sample was peeled-off into an exceptionally smooth surface. Figure 6a,b show the transmittance spectra of FPI-c-UT and FPI-c-TH in the visible light region. Table 2 summarizes the mean transmittances of the FPI-c series films according to XFL fluence (400–750 nm).

When the XFL fluence increased from 0.85 to 5.8 J/cm^2^, the mean transmittance of the films decreased to −2.5% for FPI-c-UT, −1.9% for FPI-c, and −1.7% for FPI-c-TH. Therefore, it was observed that as the film became thinner, its transmittance decreased more dramatically with increase in the fluence. It was noted that as the FPI-c series samples became thinner, they also showed a transmittance edge shift toward the UV region. This behavior follows the theory that as the film thickness decreases, the transmission range expands toward the UV region. Therefore, it has been demonstrated that when a PI substrate with an appropriate chemical structure undergoes the XF-LO process, a very flexible and transparent electronic device can be obtained.

Figure 6c shows the TGA data of the FPI-c series films after undergoing the XF-LO process. All samples were peeled-off by 0.85 J/cm^2^ XFL fluence, and the same mass (5 mg) of them was analyzed at a heating rate of 20 °C/min in a nitrogen gas atmosphere. The T5 wt% were 514 °C, 532 °C, and 538 °C for FPI-c-UT, FPI-c, and FPI-c-TH, respectively. As the thickness of the films increased, the heat resistances also increased. However, the FPI-c-UT showed no critical decrease in heat resistance despite its ultrathinness.

### 3.5. Investigation of the Surface Characteristics of the Released PI Films

**Figure 7:**
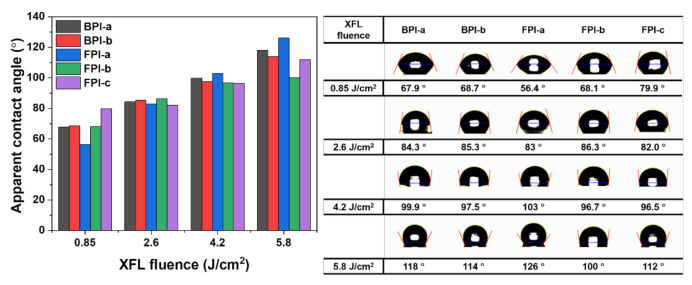
Apparent contact angle of water droplets on the released surface films with various applied XFL fluences.

In general, the behavior of liquid droplets on a solid surface may vary depending on the surface energy, which is determined by its chemical and physical (i.e., roughness) properties [25]. For instance, in the field of laser surface treatment, either the photochemical ablation mechanism causes the surface to be oxidized [26,27], or the photothermal ablation mechanism causes the surface to be carbonized [28,29]. The hydrophobic carbonized surface either undergoes chemical decomposition or physical deformation, leading to an uneven structure, which influences the apparent contact angle of the films. Figure 7 shows the apparent contact angle of the released PI films. The apparent contact angle is the angle between the apparent solid surface and the tangent to the liquid-fluid interface. [30] However, most practical systems do not possess ideal symmetry. Thus we measured the angle at both ends and defined its average value as the apparent contact angle.

**Figure 8:**
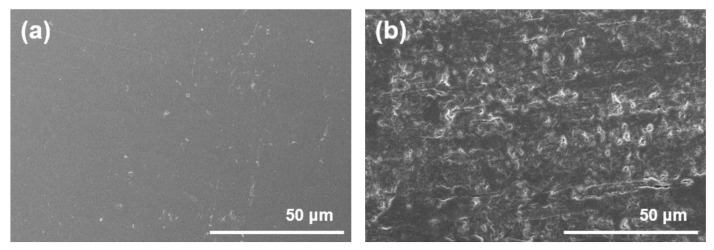
FE-SEM images of the surface of the FPI-a film treated with the XFL fluence of (**a**) 0.85 and (**b**) 5.8 J/cm^2^.

All samples confirmed that the apparent contact angle increases as the XFL fluence increases. In particular, the contact angle of the FPI-a film was 56.4° at 0.85 J/cm^2^ and 126° at 5.8 J/cm^2^, showing the highest increase. It is believed that the converted heat induced structural deformation and thermal decomposition near the FPI-a film surface. The intensified haze and surface irregularities of this sample after treatment with the highest XFL fluence of 5.8 J/cm^2^ appeared clearly and compared with the intact surface in the FE-SEM image (Figure 8). This surface deterioration was also the reason of drastically decreased transmittance in the aforementioned chapter. Therefore, the optimum XFL fluence necessary for the lift-off of a high quality surface substrate should be investigated.

### 3.6. Investigation of Dielectric Properties of Peeled-off Polyimide Films

**Figure 9:**
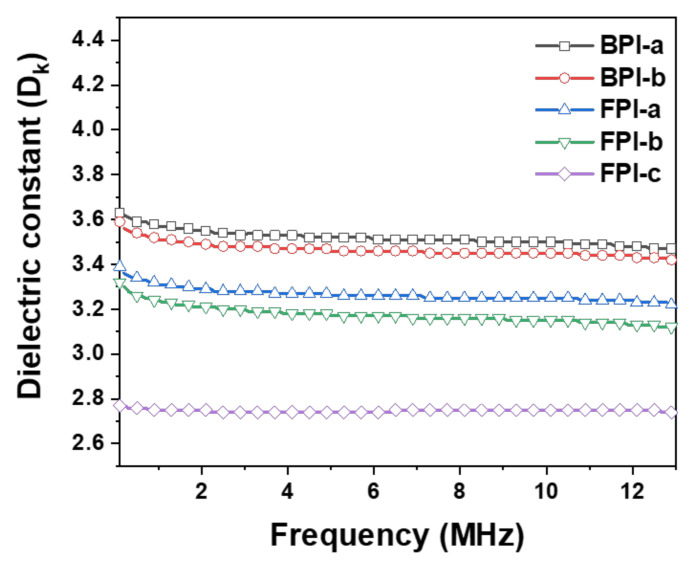
Dielectric constants of prepared PI films in the frequency range of 100 kHz to 13 MHz.

Next-generation electronic devices are continuously being miniaturized and integrated. Accordingly, problems such as power loss, cross talk between lines, and resistance-capacitance time delay (RC delay) can occur. The development of low dielectric constant materials is one of the most important factors for state-of-the-art electronic applications. The dielectric constant (*D_k_*) of a material is defined by the Clausius–Mosotti equation as follows [31]:

$$D_{k}=\frac{1+2\left(\frac{P_{m}}{V_{m}}\right)}{1-\left(\frac{P_{m}}{V_{m}}\right)}$$

where *P_m_* and *V_m_* are the molar polarizability and molar volume of the atomic groups, respectively. To achieve a low dielectric constant, the molar polarizability should be reduced and the molar volume of the atomic groups should be increased. For example, the –CF_3_ functional group, which has a high molar volume and low polarizability, can be introduced into the PI chain [32]. Figure 9 shows the dielectric constants of the peeled-off PI films at various frequencies. All the measured dielectric constants decreased as the applied frequency increased. At 13 MHz, BPI-a and BPI-b, without –CF_3_ functional groups, had dielectric constants 3.46, and 3.42, respectively. At this same frequency, FPI-a and FPI-b, with the –CF_3_ functional groups, had dielectric constants 3.22, and 3.12, respectively. The FPI-b sample, which contained an ether linkage, exhibited a lower dielectric constant than FPI-a. This is attributed to the increased free volume. In particular, FPI-c, which contained –CF_3_ functional groups in both the anhydride and diamine parts, exhibited the lowest dielectric constant value of 2.74. All peeled-off samples exhibited inherently low dielectric constants even after the XF-LO process.

## 4. Conclusions

The lift-off behavior of PI films with various chemical structures was investigated for use in the new XF-LO process, a next-generation lift-off technology that will replace the current dominant laser lift-off process. The PI substrates used in the experiment were designated as non-fluorine PI series or fluorine PI series, and synthesized in a manner suitable to their chemical structures. Based on the characteristics of the peeled-off PI films, the XF-LO process mechanism was identified as photothermal decomposition. This occurs by thermal conduction via light-to-heat conversion. The synthesized PI films treated with the XF-LO process exhibited various thermal, optical, dielectric, and surface characteristics depending on their chemical structures. The fluorine PI series exhibited the more stable lift-off behavior, while the non-fluorine PI series showed the better thermal stability. The over-exposed XFL fluence induced the intensified haze and surface irregularities of PI film with a rigid rod chemical structure. The PI molecules with high concentrations of –CF_3_ functional groups and kinked chemical structures demonstrated the most promising peeling properties, optical transparencies, and dielectric constants. In particular, an ultra-thin PI substrate (6 μm) was successfully fabricated and showed potential for use in next-generation flexible electronics.

## Figures and Tables

**Table 1:** Mean optical transmittance of films by various XFL fluence.

Films	Mean T (%, 500–750 nm)			
	XFL Fluence (J/cm^2^)			
	0.85	2.6	4.2	5.8
BPI-a	90.2	85.6	81.0	71.6
BPI-b	87.1	84.3	79.8	77.8
FPI-a	89.5	86.6	82.1	46.2
FPI-b	88.3	88.1	86.3	85.3
FPI-c	92.3	92.1	90.5	89.9

**Table 2:** Mean optical transmittance of FPI-c films by thickness and XFL fluence.

Films	Mean T (%, 400–750 nm)			
	XFL Fluence (J/cm^2^)			
	0.85	2.6	4.2	5.8
FPI-c-UT (6μm)	91.7	90.7	90.0	89.2
FPI-c (12μm)	91.6	91.4	90.3	89.7
FPI-c-TH (30μm)	92.3	92.1	91.6	90.6

## Supplementary Materials

The following are available online at https://www.mdpi.com/2073-4360/13/4/546/s1, Figure S1: FT-IR spectra and EDX data of prepared FPI film series.

## References

[1] Mativenga M., Geng D., Kim B., Jang J. (2015). Fully Transparent and Rollable Electronics. ACS Appl. Mater. Interfaces.

[2] Shi Y., Wang C., Yin Y., Li Y., Xing Y., Song J. (2019). Functional Soft Composites As Thermal Protecting Substrates for Wearable Electronics. Adv. Funct. Mater..

[3] Kang Z., Zhang Y., Zhou M. (2019). AgNPs@CNTs/Ag hybrid films on thiolated PET substrate for flexible. Chem. Eng. J..

[4] Xu H., Luo D., Li M., Xu M., Zou J., Tao H., Lin-Feng L., Wang L., Peng J., Cao Y. (2014). A flexible AMOLED display on the PEN substrate driven by oxide thin-film transistors using anodized aluminium oxide as dielectric. J. Mater. Chem. C.

[5] Hanada T., Negishi T., Shiroishi I., Shiro T. (2010). Plastic substrate with gas barrier layer and transparent conductive oxide thin film for flexible displays. Thin Solid Films.

[6] Lei P.-H., Hsu C.-M., Fan Y.-S. (2013). Flexible organic light-emitting diodes on a polyestersulfone (PES) substrate using Al-doped ZnO anode grown by dual-plasma-enhanced metalorganic deposition system. Org. Electron..

[7] Ghosh D.S., Chen T.L., Mkhitaryan V., Pruneri V. (2014). Ultrathin Transparent Conductive Polyimide Foil Embedding Silver Nanowires. ACS Appl. Mater. Interfaces.

[8] Song C.-H., Han C.J., Ju B.-K., Kim J.-W. (2016). Photoenhanced Patterning of Metal Nanowire Networks for Fabrication of Ultraflexible Transparent Devices. ACS Appl. Mater. Interfaces.

[9] Jeong G., Koo D., Seo J., Jung S., Choi Y., Lee J., Park H. (2020). Suppressed Interdiffusion and Degradation in Flexible and Transparent Metal Electrode-Based Perovskite Solar Cells with a Graphene Interlayer. Nano Lett..

[10] Bian J., Zhou L., Wan X., Liu M., Zhu C., Huang Y., Yin Z. (2019). Experimental study of laser lift-off of ultra-thin polyimide film for flexible electronics. Sci. China Technol. Sci..

[11] Bian J., Zhou L., Yang B., Yin Z., Huang Y. (2020). Theoretical and experimental studies of laser lift-off of nonwrinkled ultrathin polyimide film for flexible electronics. Appl. Surf. Sci..

[12] Kim Y., Noh Y., Park S., Kim B.-K., Kim H.J. (2020). Ablation of polyimide thin-film on carrier glass using 355 nm and 37 ns laser pulses. Int. J. Heat Mass Transf..

[13] Cozzens R.F., Fox R.B. (1978). Infrared laser ablation of polymers. Polym. Eng. Sci..

[14] Brannon J.H., Lankard J.R., Baise A.I., Burns F.C., Kaufman J.H. (1985). Excimer laser etching of polyimide. J. Appl. Phys..

[15] Dyer P.E., Jenkins S.D., Sidhu J. (1986). Development and origin of conical structures on XeCl laser ablated polyimide. Appl. Phys. Lett..

[16] Taylor R.S., Singleton D.L., Paraskevopoulos G. (1987). Effect of optical pulse duration on the XeCl laser ablation of polymers and biological tissue. Appl. Phys. Lett..

[17] Lee S.I., Jang S.H., Han Y.J., Lee J.Y., Choi J., Cho K.H. (2020). Xenon Flash Lamp Lift-Off Technology without Laser for Flexible Electronics. Micromachines.

[18] Okada T., Ishige R., Ando S. (2018). Effects of chain packing and structural isomerism on the anisotropic linear and volumetric thermal expansion behaviors of polyimide films. Polym..

[19] Li K., Liu Y., Zheng H., Huang G., Li G. (2011). Enhanced gas-condensate production by wettability alteration to gas wetness. J. Pet. Sci. Eng..

[20] Tapaswi P.K., Ha C.-S. (2019). Recent Trends on Transparent Colorless Polyimides with Balanced Thermal and Optical Properties: Design and Synthesis. Macromol. Chem. Phys..

[21] Yi C., Li W., Shi S., He K., Ma P., Chen M., Yang C. (2020). High-temperature-resistant and colorless polyimide: Preparations, properties, and applications. Sol. Energy.

[22] Choi I.H., Sohn B., Chang J.-H. (2010). Synthesis and characterization of transparent copolyimide films containing CF3 groups: Comparison with copolyimide nanocomposites. Appl. Clay Sci..

[23] Go H., Han E.-M., Kang M.H., Kim Y.-H., Yun C. (2017). The coated porous polyimide layers for optical scattering films. AIMS Mater. Sci..

[24] Yang Y., Park J.H., Jung Y., Lee S.W., Kil Park S., Kwon S. (2016). Effect of fluorination on haze reduction in transparent polyimide films for flexible substrates. J. Appl. Polym. Sci..

[25] Oliveira V.M., Nunes B., Vilar R. (2010). Wetting response of KrF laser ablated polyimide surfaces. Nucl. Instrum. Methods Phys. Res. B.

[26] Wang Z., Zheng H., Lim C.P., Lam Y. (2009). Polymer hydrophilicity and hydrophobicity induced by femtosecond laser direct irradiation. Appl. Phys. Lett..

[27] Lippert T. (2005). Interaction of Photons with Polymers: From Surface Modification to Ablation. Plasma Process. Polym..

[28] Brezini A., Zekri N. (1994). X-ray photoelectron spectroscopy analysis of polyimide films modified by ultraviolet pulsed laser radiation at 193 nm. J. Appl. Phys..

[29] Lippert T., Ortelli E., Panitz J.-C., Raimondi F., Wambach J., Wei J., Wokaun A. (1999). Imaging-XPS/Raman investigation on the carbonization of polyimide after irradiation at 308 nm. Appl. Phys. A.

[30] Wolansky G., Marmur A. (1999). Apparent contact angles on rough surfaces: The Wenzel equation revisited. Colloids Surf. A Physicochem. Eng. Asp..

[31] Lv P., Dong Z., Dai X., Wang H., Qiu X. (2018). Synthesis and properties of ultralow dielectric porous polyimide films containing adamantane. J. Polym. Sci. A Polym. Chem..

[32] Tao L., Yang H., Liu J., Fan L., Yang S. (2009). Synthesis and characterization of highly optical transparent and low dielectric constant fluorinated polyimides. Polymer.

