# Supplementary material for: Investigation of the Chemical Structure of Ultra-Thin Polyimide Substrate for the Xenon Flash Lamp Lift-off Technology
Source: Polymers (Basel). 2021 Feb 12;13(4):546. doi: 10.3390/polym13040546 (PMC7918077; doi:10.3390/polym13040546)
Supplement: Supplementary file 1 [file polymers-13-00546-s001.pdf]

polymers

Article

Investigation of the Chemical Structure of Ultra-Thin Polyimide Substrate for the Xenon Flash Lamp Lift-off Technology

Seong Hyun Jang [1,3], Young Joon Han [2], Sang Yoon Lee [1,3], Geonho Lee [1,4], Jae Woong Jung [3,*], Kwan Hyun Cho [2,*] and Jun Choi [1,*]

1 Human Convergence Technology R&D Department, Korea Institute of Industrial Technology (KITECH), Ansan 15588, Republic of Korea
2 Manufacturing Process Platform R&D Department, Korea Institute of Industrial Technology (KITECH), Ansan, 15588, Republic of Korea
3 Department of Advanced Material Engineering for Information & Electrices, Kyunghee University, Yongin, 17104, Republic of Korea
4 Department of Chemical Engineering, Kyunghee University, Yongin, 17104, Yongin, Republic of Korea
* Correspondence: wodndwjd@khu.ac.kr (J.W.J.); khcho@kitech.re.kr (K.H.C.); skywork1@kitech.re.kr (J.C.) Tel.: +82 31 8040 6256 (J.C.)

Citation: Jang, S.H.; Han, Y.J.; Lee, S.Y.; Lee, G.; Jung, J.W.; Cho, K.H.; Choi, J. Investigation of the Chemical Structure of Ultra-Thin Polyimide Substrate for the Xenon Flash Lamp Lift-off Technology. *Polymers* **2021**, *13*, 546. https://doi.org/10.3390/polym13040546

Academic Editor: Toshihiko Matsumoto

Received: 21 January 2021
Accepted: 8 February 2021
Published: 12 February 2021

Publisher's Note: MDPI stays neutral with regard to jurisdictional claims in published maps and institutional affiliations.

Copyright: © 2021 by the authors. Submitted for possible open access publication under the terms and conditions of the Creative Commons Attribution (CC BY) license (http://creativecommons.org/licenses/by/4.0/).

1. Supplementary Materials

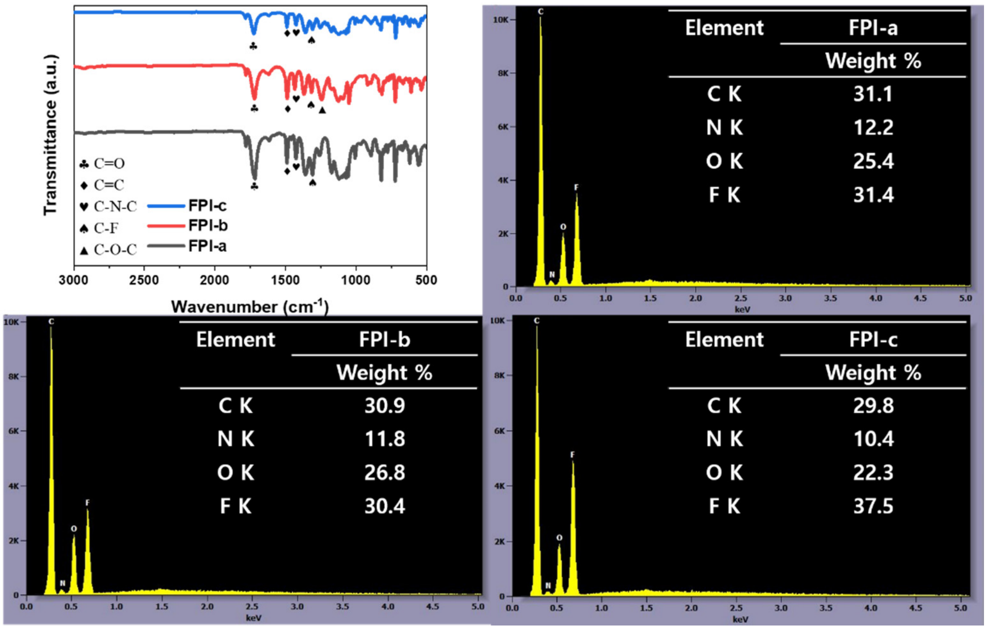

Figure S1. FT-IR spectra and EDX data of the prepared FPI film series.
